# Automatic segmentation of inner ear on CT-scan using auto-context convolutional neural network

**DOI:** 10.1038/s41598-021-83955-x

**Published:** 2021-02-23

**Authors:** Raabid Hussain, Alain Lalande, Kibrom Berihu Girum, Caroline Guigou, Alexis Bozorg Grayeli

**Affiliations:** 1 ImViA Laboratory, University of Burgundy Franche Comte, Dijon, France; 2 Medical Imaging Department, University Hospital of Dijon, Dijon, France; 3 Otolaryngology Department, University Hospital of Dijon, Dijon, France

**Keywords:** Tomography, Medical imaging, Medical research

## Abstract

Temporal bone CT-scan is a prerequisite in most surgical procedures concerning the ear such as cochlear implants. The 3D vision of inner ear structures is crucial for diagnostic and surgical preplanning purposes. Since clinical CT-scans are acquired at relatively low resolutions, improved performance can be achieved by registering patient-specific CT images to a high-resolution inner ear model built from accurate 3D segmentations based on micro-CT of human temporal bone specimens. This paper presents a framework based on convolutional neural network for human inner ear segmentation from micro-CT images which can be used to build such a model from an extensive database. The proposed approach employs an auto-context based cascaded 2D U-net architecture with 3D connected component refinement to segment the cochlear scalae, semicircular canals, and the vestibule. The system was formulated on a data set composed of 17 micro-CT from public Hear-EU dataset. A Dice coefficient of 0.90 and Hausdorff distance of 0.74 mm were obtained. The system yielded precise and fast automatic inner-ear segmentations.

## Introduction

**Figure 1:**
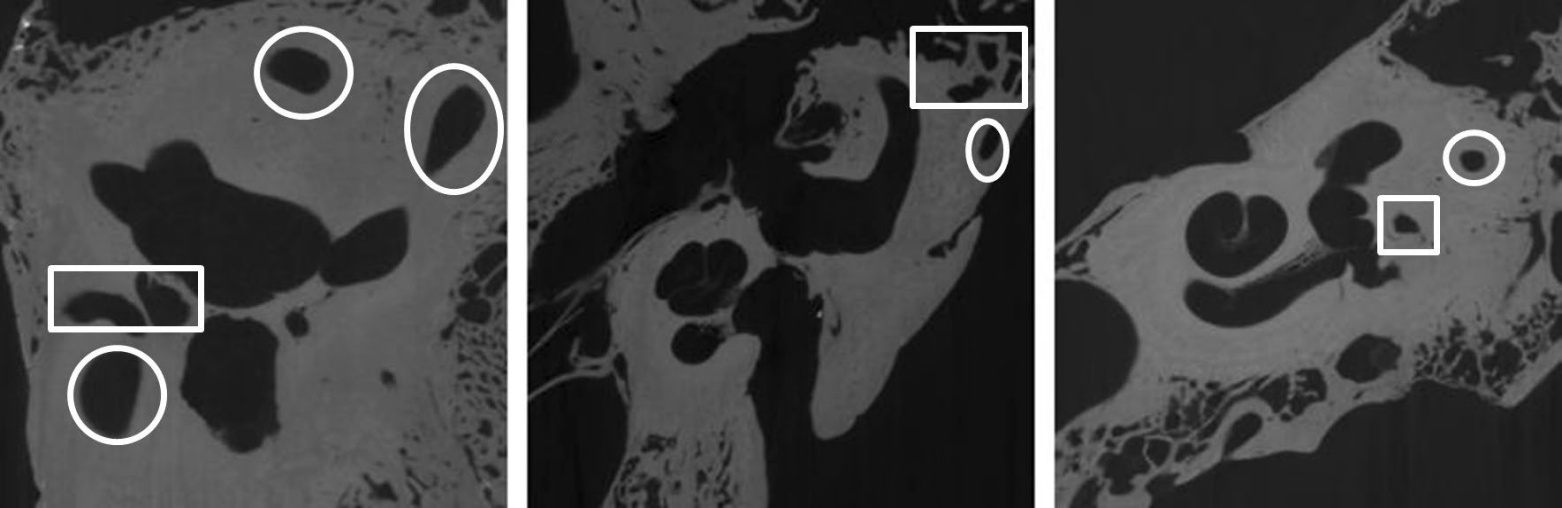
Difficulties in segmenting similarly looking structures in inner ear CTs (using independent image slices). Structures that are part of the inner ear are highlighted with a circle whereas the structures not part of the inner ear are highlighted with a rectangle.

Temporal bone CT-scan is largely used for diagnostic and surgical preplanning in diseases involving the inner ear such as hearing loss and balance disorders^1^. In routine practice, this technique offers a series of 2D images which are browsed back and forth by the practitioner to mentally deduce 3D information and to this end 3D reconstructions have been applied to training and surgical planning^2^. On independent 2D image slices, multiple structures can be confounded with inner ear and the mental reconstruction of inner ear structures can be difficult (Fig. 1). Commercially available volume rendering techniques may provide useful information about large structures such as large vessels or lungs^3,4^ but are not accurate and robust enough for otological applications because the small size of the structures and large variations in background intensity values around the inner ear boundaries may generate artefacts^5^. Nevertheless, 3D anatomical information is crucial before an ear surgery to predict difficulties and to adapt instrumentation and approach^6^. For example, when an electrode array is inserted into the cochlea during a cochlear implantation, the information about the anatomy of the cochlea (e.g. malformations, lumen obstruction or narrowing) or its size influences the choice of the array^7^. Inner ear has a complex 3D anatomy and is surrounded by critical structures like facial nerve and blood vessels, with only few visible landmarks during surgery^8–10^. Moreover, its anatomy is subject to great inter individual variability justifying even more the use of preoperative CT-scan^10–12^.

Automated or aided tridimensional reconstruction of anatomical regions including the temporal bone and the inner ear can be conducted by commercially available softwares^13–16^. The main drawback of these generic softwares is the need for interactions and significant anatomical expertise to conduct the segmentation, which could make the work tedious and time-consuming in clinical practice. Softwares specific to temporal bone often use basic algorithms such as region growing, boundary detection and thresholding to segment the cochlea^17–20^. This often leads to incomplete segmentation since the identification of inner ear structures solely based on intensity generates multiple errors at each slice and generally yields an aberrant final 3D image. As an alternative, inner ear segmentations are often carried out manually during a tedious and time-consuming process^21^. Several projects have proposed solutions via semi-automatic algorithms such as 3D-level sets and interactive contour algorithms^22–24^. However, they still require user interaction and introduce human error into the system. Fully automatic algorithms based on active statistical shape modelling have been applied to cochlear segmentation^25,26^, but to attain an accurate statistical shape in different scenarios, a large amount of annotated data would be required^27^. Other proposed solutions such as atlas-based frameworks^11,12^ and iterative random-walks algorithm with shape prior integration produced encouraging results for cochlea segmentation but are computationally expensive^28,29^. Moreover, with shape priors and atlas-based methods, segmentation might fail if the analysed image diverges from the average shape model, and this is often the case in malformations. Furthermore, these methods can reach theirs limits with clinical CTs at low resolutions which do not include elaborative details on the cochlea.

Deep-learning strategies have gained immense popularity in segmentation tasks where they outperform conventional approaches^30^. In the medical field, large image sizes and limited availability of expert annotations are the main challenges of deep-learning algorithms which have been recently tackled^31,32^. To optimize performances, U-Net has been the most popular base-architecture algorithm in this field for several years^32^. This architecture allows processing a considerable amount of data with a relatively low computational cost and without loss of detail. It has been named after the “U” shape of its architecture: the system downsamples the data while extracting higher level features for a faster processing, and upsamples it again by integrating stored details to deliver a high-quality result^33^.

To deal with a limited set of data, patch-based learning is effective. This method consists of selecting and analysing patches of relevant features inside an image to classify it instead of using the entire image^34,35^. This method was developed to reduce the computational burden in selected scenarios, but inherently, it relies on local features instead of using the contextual information. In particular, processing large stacks of high-resolution CT-scan slices (> 1000) by this method is still computationally expensive.

Multi-view algorithms have been proposed to integrate contextual information into the segmentation framework. These algorithms segment structures independently from each orthogonal view and then combine the output using either majority voting, connected component analysis, sigmoid/softmax output scores or FCN output layer^36–38^. However, these architectures do not take the outputs of other views into account in the segmentation architectures. Auto-context is an alternative algorithm that combines low-level appearance features to high-level shape information extracted from all three orthogonal planes^39^. This architecture was conceived as an alternative to cascaded network schemes in which the output of one network is fed to a second network to incorporate spatial context^40^. By the combination of these two different approaches, auto-net was developed^41^. This algorithm fuses the posterior distribution of labels with image features, and it has shown encouraging results in segmenting brain images^41^. However, it is also performed on image patches and is an iterative algorithm.

We hypothesized that by using deep-learning architectures in an auto-context framework, and by avoiding patch-based classifiers and relatively extensive iterative schemes which are computationally expensive, we could obtain a rapid and precise segmentation of the inner ear from a highly-detailed data set (micro-CT scans).

The aim of this work was to develop and evaluate a fully automatic framework for segmentation of the inner ear structures from micro-CT images. We present an auto-context cascaded convolutional neural network using a three-stage orthogonal U-Net model with connected component extraction after each stage to robustly segment inner ear structures from the micro-CT images.

## Material and methods

### Dataset preprocessing

The Hear-EU cochlear public dataset was chosen for this project^42^. It consists of micro CT-scans from 17 human temporal bone specimens. The ground truth labels, manually delineated by an expert neuroradiologist on each slice, consisted of cochlear scalae, semicircular canals, and the vestibule. The images were acquired at 16.3 μm for 13 specimens and 19.5 μm voxel resolutions for the remaining 4. The original volume size of the CT-scans ranged from 618 × 892 × 600 to 1500 × 1500 × 1500. CT-scans were resampled to a fixed size of 256 × 256 × 256 voxels (using spline interpolation), corresponding to 4352 image slices in the dataset for computational and memory requirements. The ground truth labels were not aligned with the raw CT-scan images in 5 specimens and these data were manually aligned with the CT-scan data. The intensity values were zero-centered and normalized by the standard deviation of the training dataset in each experiment and for each cross-validation fold. No parallelization was employed for data processing.

### Auto-cascaded net (AutoCasNet)

**Figure 2:**
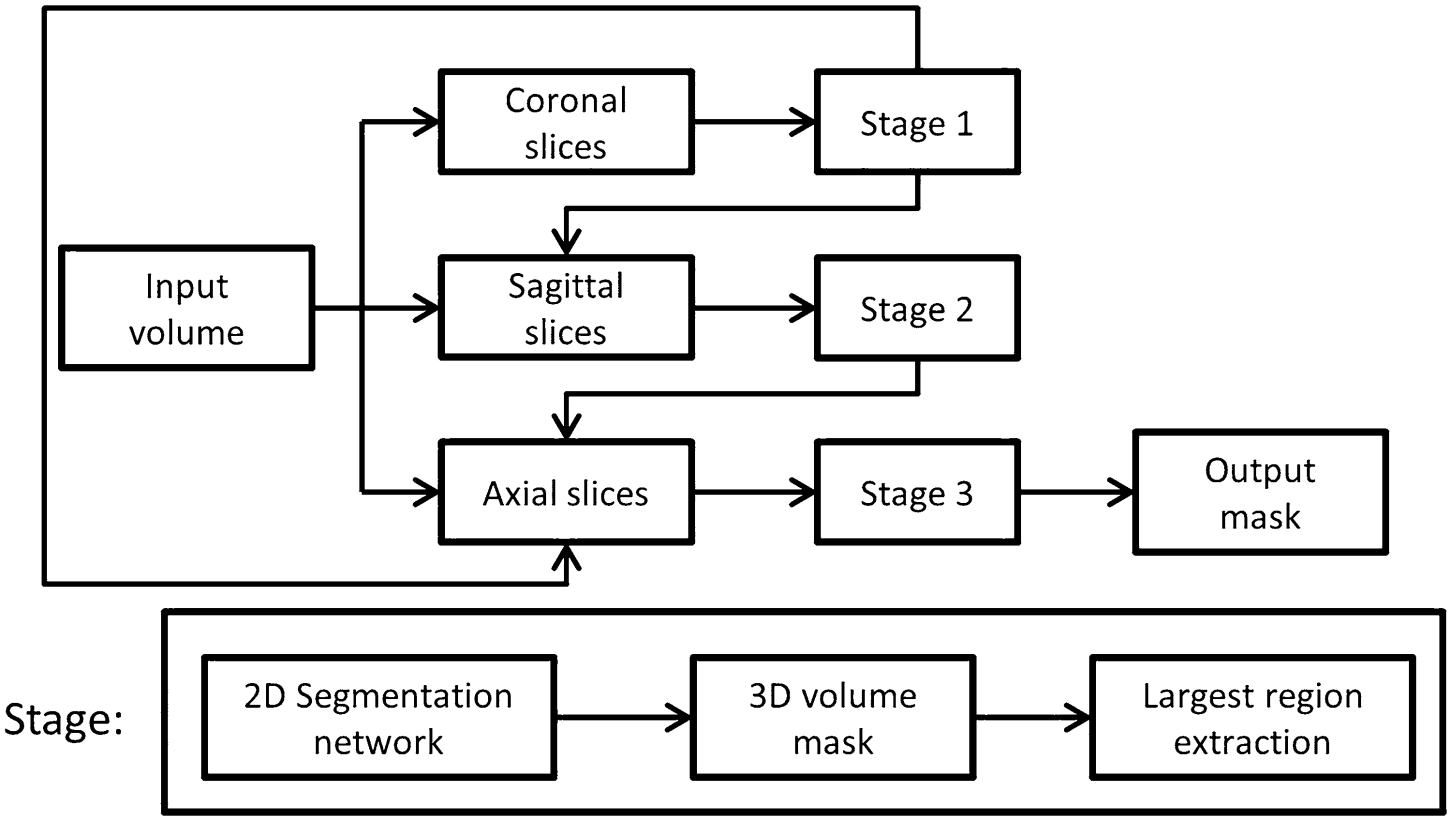
Workflow of the proposed framework. The framework is compatible with any 2D segmentation architecture. In this article, the following 2D segmentation networks were used: U-Net, Residual U-Net and SEU-Net.

The proposed methodology (Fig. 2) employed a 2D segmentation algorithm to segment each image slice individually in a three-stage cascaded framework to incorporate 3D information. The system began by partitioning the input volumes into axial, coronal and sagittal slices. In the first stage, coronal slices of each CT were fed to a 2D segmentation architecture. The output was then assembled to form a segmented 3D volume. The largest connected component region was extracted from this volume and partitioned into sagittal slices. This output of the first stage was fed to the second stage as binary masks and additional image channels along with their corresponding image slices from the original CT volume. Again, a segmented 3D volume was obtained and fed to the third stage. Finally, in the third stage, axial slices from all previous stages and original CT volume were used as input. The output of this last stage was used as the final system output.

**Figure 3:**
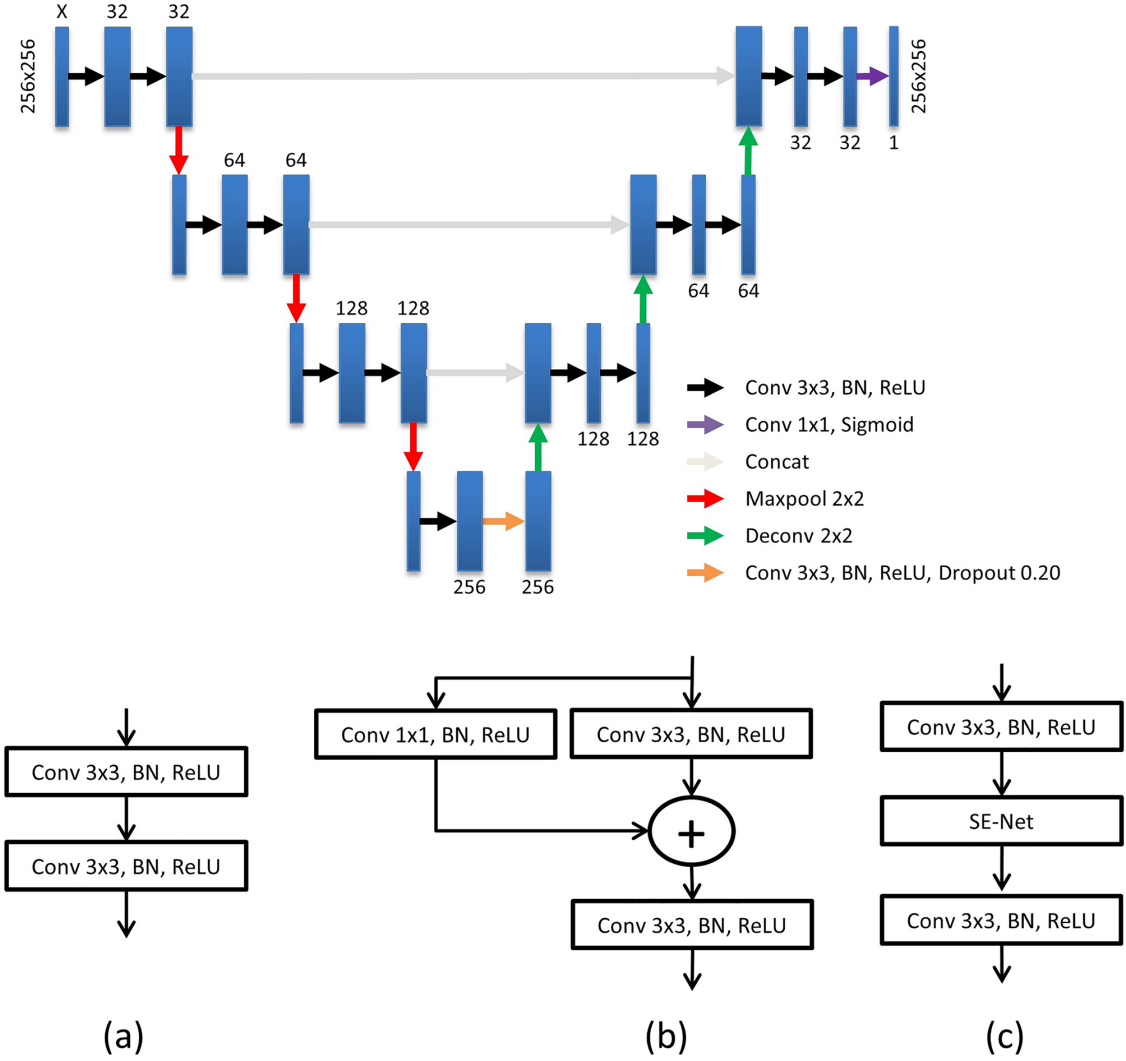
2D segmentation architectures. **(a)** U-Net, **(b)** residual U-Net, **(c)** SEU-Net with constant ratios X = {1,2,3} in stages {1,2,3} respectively. See Fig. 2 for details.

The architecture of the 2D segmentation network was based on encoder-decoder style networks (Fig. 3). The network took a 256 × 256 image slice with ‘X’ image channels as input and output the image mask, where ‘X = 1, 2 or 3’. Every step on the contracting stage consists of two consecutive blocks each having a 3 × 3 convolutional layer followed by batch normalization (BN) and rectified linear unit (ReLU) activation. This was followed by a 2 × 2 max pooling layer. The expanding stage was composed of a similar block preceded by deconvolutional layer and concatenation. Finally, we added a dropout regularization with a 20% dropout rate in the bottleneck layer to avoid overfitting. The output layer was a 1 × 1 convolutional layer with sigmoid activation function.

### Training

The network was trained using individual slices of the input volumes. The weights were updated using Adam optimizer with a learning rate of 0.0001. The training was carried out with a batch size of 20 for maximum 200 epochs with early-stopping criteria in each stage, where the best model on training dataset (with the lowest training loss) was checkpointed and stored for testing. The training was carried out with a batch size of 20 for 200 epochs in each stage, where the best model was checkpointed and stored for testing. The binary cross entropy was used as the cost function. The first network was trained until convergence and its output was post-processed and fed into the second network as input for training and similarly for the last stage. The model was implemented using Keras and TensorFlow libraries on a standard Intel i7 computer with 32 GB RAM and a dedicated GPU (Nvidia Titan X processor, 12 GB RAM) and validated using a fourfold cross validation approach with all slices belonging to a patient kept in the same fold. The fourfold evaluation, with hold-out test sets, was performed to reduce the influence of biases in the quantitative analysis^43^. The dataset included in this study was obtained from two different scanners and acquisition protocols and was randomly placed into the different folds. During the evaluation, different combinations of the orthogonal axes were also tested to determine the optimum ordering of orientation. The code is available at https://github.com/raabid236/AutoCasNet.

As comparison, the runtime of the framework was also tested on a standard Intel Xeon E5-2609 computer with 16 GB RAM.

### 2D-segmentation architectures

Three segmentation architectures were compared. Each of them was used independently (on the best performing slice orientation) and as the basic segmentation algorithm in our proposed framework:

1. U-Net: Our design was adapted from the original paper^33^ with batch normalization integration and a change in number of layers and filter sizes (Fig. 3). Dropout regularization was performed on the bottleneck layer. A post-processing step (largest region extraction) was also applied to refine the output. The basic block of the architecture is presented in detail in Fig. 3a.
2. Residual U-Net (ResU-Net): ResU-Net followed the same architecture as in Fig. 3 but incorporated residual blocks^44,45^ instead of convolution blocks in down- and up-sampling layers. The residual blocks consisted of 3 × 3 and 1 × 1 convolutions summed together (Fig. 3b). Like U-Net, a post processing step was also integrated.
3. Squeeze and Excitation U-Net (SEU-Net): SEU-Net also followed the same architecture as in Fig. 3 but incorporated squeeze and excitation blocks (with constant ratio in all layers)^46^ along with convolution blocks in down- and up-sampling layers (Fig. 3c). Like previous networks, a post processing step was also integrated.

### Evaluation

The evaluation was based on the following volume and distance-based metrics: Dice coefficient ($\frac{2|X\cap Y|}{\left|X\right|+|Y|}$), Jaccard index ($\frac{|X\cap Y|}{|XUY|}$), segmentation accuracy, precision ($\frac{|X\cap Y|}{|X|}$), recall ($\frac{|X\cap Y|}{|Y|}$), specificity, area under receiver operating characteristic (ROC) curve and 3D Hausdorff distance ($\mathrm{max}\{{sup}_{x\in X}{inf}_{y\epsilon Y}d\left(x,y\right),{sup}_{y\in Y}{inf}_{x\epsilon X}d(x,y)\}$), where X and Y are the segmentation results and the groundtruth respectively^47^. A Wilcoxon signed-rank test was also performed on the segmentation outputs to analyze the impact of AutoCasNet frameworks. A p-value < 0.05 was considered as significant.

## Results

**Figure 4:**
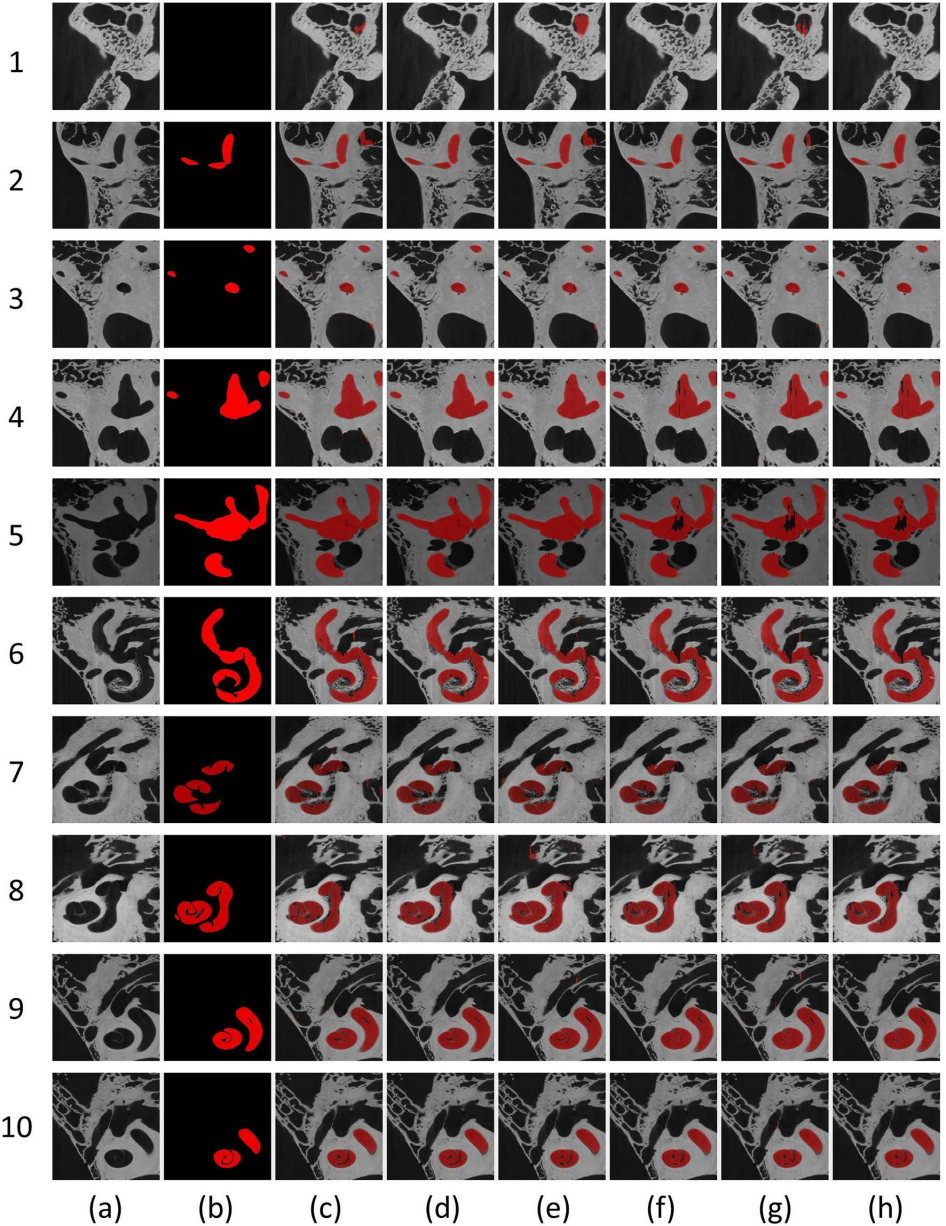
Qualitative segmentation results. **(a)** Original input image slices, **(b)** groundtruth labels, **(c)** U-Net, **(d)** AutoCasU-Net, **(e)** ResU-Net, **(f)** AutoCasResU-Net, **(g)** SEU-Net, **(h)** AutoCasSEU-Net.

**Figure 5:**
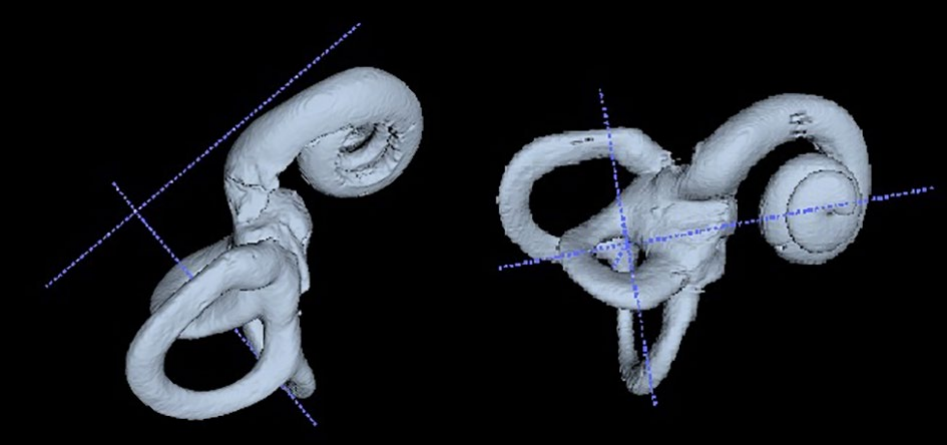
Different viewpoints of 3D reconstruction of the inner ear with the cochlea from the AutoCasU-Net segmentation output. ITK-SNAP^65^ (v 3.4.0, http://www.itksnap.org/) was used for the 3D visualisation of the segmentation output.

All networks were trained from scratch until the training error reached a plateau state. The dropout regularization helped to prevent overfitting. Table 1 depicts the quantitative results of the segmentation. In general, AutoCasNet based networks outperformed their corresponding state of the art networks^48^ in most of the evaluation metrics for all the patients in the dataset with AutoCasU-Net yielding the best segmentation. The largest improvement was seen in case of SEU-Net. Moreover, during the experiments similar results were obtained when the order of the slice orientations was changed, ensuring repeatability. Figure 4 depicts the qualitative segmentation results on different image slices. The Wilcoxon signed-rank test revealed a significant improvement in most test cases. The 3D reconstruction of the segmentation output was anatomically realistic with smooth surfaces and very small irregularities (Fig. 5).

**Table 1:** Quantitative inner ear segmentation results based on Dice coefficient (Dcc), Jaccard index (JI), accuracy (Acc), precision (Pre), recall (Rec), specificity (Spe), area under ROC curve (AUC) and Hausdorff distance (HD).

	Dcc	JI	Acc	Pre	Rec	Spe	AUC	HD (mm)
U-Net	0.884 ± 0.076	0.801 ± 0.116	0.990 ± 0.008	0.910 ± 0.070	**0.867 ± 0.102**	0.996 ± 0.004	**0.931 ± 0.052**	0.773 ± 0.273
AutoCasU-Net	0.900 ± 0.070*	0.825 ± 0.110*	0.992 ± 0.008*	0.949 ± 0.051*	0.863 ± 0.108	0.998 ± 0.003*	0.931 ± 0.054*	0.738 ± 0.289*
ResU-Net	0.869 ± 0.098	0.781 ± 0.139	0.980 ± 0.011	0.901 ± 0.071	**0.852 ± 0.137**	0.995 ± 0.005	**0.920 ± 0.069**	0.796 ± 0.300
AutoCasResU-Net	**0.871 ± 0.126**	**0.789 ± 0.165**	0.990 ± 0.011*	0.942 ± 0.056*	0.832 ± 0.173	0.997 ± 0.004*	**0.920 ± 0.086**	0.781 ± 0.308*
SEU-Net	0.778 ± 0.144	0.656 ± 0.179	0.979 ± 0.019	0.812 ± 0.154	0.753 ± 0.152	0.990 ± 0.012	0.871 ± 0.078	1.003 ± 0.324
AutoCasSEU-Net	0.839 ± 0.107*	0.736 ± 0.142*	0.988 ± 0.008*	0.925 ± 0.077*	**0.792 ± 0.159**	0.996 ± 0.006*	**0.894 ± 0.079**	**0.897 ± 0.281**

The results for the baseline networks are for axial viewpoints (0.2–1.1% better average dice coefficient performances obtained from this view compared to coronal or sagittal orientations; data not shown). The best metrics for each comparison are shown in bold. The results are an average of the 17 3D μCT volumes. * p < 0.05, Wilcoxon signed-rank test.

In the comparative evaluation, we noted that when contextual information (intermediate outputs from previous stages) was not taken into account, incomplete and unwanted regions that have similar 2D features were also segmented (Fig. 4c,e,g). The proposed framework yielded refined output masks which were coherent with the ground truth labels for all the proposed basic architectures. However, as we can observe from rows 5 and 6 in Fig. 4, the entire region was not segmented in some slices. From visual inspection of 400 random slices, it was found that 10.5% of the slices contained such fragmented segmentation labels. Due to the spiral shape of the structures with small gaps between regions, smoothing and morphological operators were not considered to deal with such fragmented regions.

Segmentation of one 3D volume took 11 s on the GPU and 240 s on the conventional desktop computer. This time included loading the resampled data and the model and predicting the segmentation output.

## Discussion

In this study, we presented a fully automatic neural network for segmenting inner ear structures from micro-CT images. The system yielded a precise 3D reconstruction in a few seconds on a computer equipped with a GPU. A PC without a GPU produced the result in less than 5 min. The precision and the rapidity of this program are compatible with its use in routine practice.

Although the segmentation performance might increase with the integration of contextual information through 3D architectures or shape prior modelling as a post-processing step^49,50^ due to the limited size of the dataset and the large volume size, these options were excluded and a 2D architecture that processed each slice individually was adopted instead. Although patch-based 3D approaches can reduce the high-memory requirements, such approaches are still computationally expensive and they do not incorporate global context which may lead to incomplete segmentations. Another advantage of using slices instead of whole volumes was to extend the dataset size to 4352 individual and independent input cases. The disadvantage of a 2D architecture was that slices do not include all the geometric information of the inner ear and targets can be more easily confounded with similar regions (Fig. 1).

To overcome this weakness, a three-stage cascaded convolutional neural network design was adopted to incorporate 3D information. It employed a 2D U-Net architecture followed by 3D largest-region extraction at each stage in an auto-context framework. We compared the performance of the proposed framework with other deep-learning architectures, and despite the limited size of the dataset, the architecture outperformed other state-of-the-art segmentation algorithms according to several conventional metrics. ResU-Net, and SEU-Net are generally considered to be improved modifications of the original U-Net^51,52^, but even with the optimal network parameters (e.g. number of layers, filter size, learning rate), they could not compete with U-Net. This advantage was also observed for AutoCasNet frameworks with the same parameters. This difference may be explained by the complex nature of inner ear anatomy and the common choice of hyperparameters in all the frameworks. Indeed, systematic comparisons of U-Net performance in the field of virus identification has shown that a judicious choice of hyperparameters for each framework are pivotal especially if a limited number of trainable weights are used^53^.

The runtime was adequate for clinical use and could be further improved with a C +  + implementation (instead of Python which is comparatively a higher-level language)^54^. The relative high standard deviation for some metrics could be due to the fact that temporal bones contain air cells that resemble inner ear cavities on many sections. These air cells have variable sizes and locations. This difficulty is inherent to the model. It could hamper the inner ear identification and the reproducibility of the procedure.

Although deep-learning-based methods were used in the AutoCasNet framework in this project, conventional 2D segmentation algorithms could replace them as the basic segmentation technique in this framework. However, conventional algorithms exhibiting good performance for cochlear segmentation have been observed to be computationally expensive^11,12,28,29^. In a future step, we propose to integrate learned prior shape models in the 2D segmentation algorithm through deep generative networks^50,55^. This would help to generate a proposal for the shape of the inner ear in a given image slice based on its CT image which can be used to refine the segmentation output.

In future, our framework can be used to build a detailed and robust inner ear model using statistical shape modelling from a high number of micro-CT segmentations^42^. Also, it will be adapted to abnormal inner ear anatomy to extend its clinical and research applications. Statistical shape modelling algorithms are often used to represent anatomical variations of target structures in a compact parametric model by first registering segmentations obtained from different patients and generating a mesh structure through deformation field generation^56,57^. This detailed statistical model can then be registered with the segmentation from a patient's clinical CT using automatically extracted anatomical points or intensity-based algorithms^58,59^. The resulting high-quality co-registered data set of the human bony labyrinth can be used to study microscopic inner ear morphology in detail, for developing efficient design of neuroprostheses and for surgical planning during minimally invasive treatment^42,58,60^.

Understanding the congenital or acquired (e.g. otosclerosis, fracture, fibrosis) abnormal inner ear anatomy via 3D reconstruction has major consequences on diagnosis, management, and surgical preplanning^61^. Although the focus of this study was to provide a 3D segmentation of inner ear structure for surgical or clinical applications, the reconstruction of complex living tissues has other applications such as developing finite-element models and 3D printing. For example, the segmentations can be used to modify an already existing finite-element model of the ear in order to estimate the behaviour of an ear with its specific anatomical characteristics, especially when considering the significant interindividual variations of the human inner ear morphology^62^. Our study is also a first step towards the automatic 3D-reconstruction of the inner ear in abnormal cases. To adapt our system to these complicated cases, specific training with CT-scans from inner ear malformations can be envisaged. Since data on malformation and other acquired abnormalities are relatively rare, artificial data augmentation and transfer learning strategies should be considered to increase the training of the network^63,64^.

## Conclusion

The AutoCasU-Net framework yielded accurate ear segmentations on micro-CT images of human ear in a few seconds. This method has the potential to be applied to routine CT-scans for diagnostic and surgical preplanning purposes.

## Data Availability

The data i.e. the micro-CTs that were used in this study are publically available at: https://www.smir.ch/objects/204388.
